# Ternary Nanocomposites Based on Oxidized Carbon Nanohorns as Sensing Layers for Room Temperature Resistive Humidity Sensing

**DOI:** 10.3390/ma14112705

**Published:** 2021-05-21

**Authors:** Bogdan-Catalin Serban, Cornel Cobianu, Octavian Buiu, Marius Bumbac, Niculae Dumbravescu, Viorel Avramescu, Cristina Mihaela Nicolescu, Mihai Brezeanu, Cristina Pachiu, Gabriel Craciun, Cristiana Radulescu

**Affiliations:** 1 National Institute for Research and Development in Microtechnologies-IMT Bucharest, 126 A Erou Iancu Str., 077190 Voluntari, Romania; cornel.cobianu@imt.ro (C.C.); niculae.dumbravescu@imt.ro (N.D.); viorela@imt.ro (V.A.); cristina.pachiu@imt.ro (C.P.); gabriel.craciun@imt.ro (G.C.); 2 Research Center for Integrated Systems, Nanotechnologies, and Carbon-Based Nanomaterials (CENASIC)-IMT, Str. Erou Iancu Nicolae 126A, 077190 Voluntari, Romania; 3 Academy of Romanian Scientists, Science, and Technology of Information Section, Ilfov Str., nr. 3, Sector 5, 050044 Bucharest, Romania; 4 Sciences and Advanced Technologies Department, Faculty of Sciences and Arts, Valahia University of Targoviste, 13 Sinaia Alley, 130004 Targoviste, Romania; cristiana.radulescu@valahia.ro; 5 Institute of Multidisciplinary Research for Science Technology, Valahia University of Targoviste, 13 Sinaia Alley, 130004 Targoviste, Romania; cristina.nicolescu@valahia.ro; 6 Faculty of Electronics, Telecommunications, and I.T., University Politehnica of Bucharest, Romania, 1–3 Iuliu Maniu Blvd., 6th district, 061071 Bucharest, Romania; scriemiceva@hotmail.com

**Keywords:** oxidized carbon nanohorns (CNHox), graphene oxide (GO), polyvinylpyrrolidone (PVP), resistive humidity sensor, p-type semiconductor, swelling

## Abstract

This paper presents the relative humidity (RH) sensing response of a resistive sensor employing sensing layers based on a ternary nanocomposite comprising graphene oxide-oxidized carbon nanohorns-polyvinylpyrrolidone (GO-CNHox–PVP), at 1/1/1, 1/2/1, and 1/3/1 w/w/w mass ratios. The sensing structure is composed of a silicon substrate, a SiO_2_ layer, and interdigitated transducers (IDT) electrodes, on which the sensing layer is deposited via the drop-casting method. The morphology and the composition of the sensing layers are investigated through scanning electron microscopy (SEM) and RAMAN spectroscopy. The RH sensing capability of each carbonaceous nanocomposite-based thin film was analyzed by applying a current between the two electrodes and by measuring the voltage difference when varying the RH from 0% to 100% in humid nitrogen. The sensors have a room temperature response comparable to that of a commercial humidity sensor and are characterized by a rapid response, excellent linearity, good sensitivity, and recovery time. The manufactured sensing devices’ transfer functions were established, and we extracted the response and recovery times. While the structures with GO/CNHox/PVP at 1/1/1 ratio (w/w/w) had the best performance in terms of relative sensibility, response time, and recovery time, the sensors employing the GO/CNHox/PVP nanocomposite at the 1/2/1 ratio (w/w/w) had the best linearity. Moreover, the ternary mixture proved to have much better sensing properties compared to CNHox and CNHox-PVP-based sensing layers in terms of sensitivity and linearity. Each component of the ternary nanocomposites’ functional role is explained based on their physical and chemical properties. We analyzed the potential mechanism associated with the sensors’ response; among these, the effect of the p-type semiconductor behavior of CNHox and GO, correlated with swelling of the PVP, was dominant and led to increased resistance of the sensing layer.

## 1. Introduction

In the last decades, relative humidity (RH) sensors have gained increasing interest due to their relevance in a wide variety of industrial, commercial, and residential applications such as building ventilation control, the medical field (respirators, incubators, sterilizers), food/beverage processing, chemical industry (high-temperature catalyst control system and furnaces dryers), pharmaceutical processing (quality control of drugs), agriculture (soil moisture control during irrigation), nuclear power reactors, meteorology, mining industry, robotics, textiles, paper industry, etc. [1,2].

Consequently, through the years, a plethora of principles and technologies have been improved or developed, each exhibiting both advantages and drawbacks in terms of sensitivity, response time, accuracy, drift, detection limit, cross-sensitivity, price, and so forth [3].

Among the types of devices used in the control and monitoring of humidity-capacitive [4], gravimetric [5], thermal conductivity sensors [6], and optical fiber [7], the resistive sensors are an attractive option. Moreover, their simplicity in construction, interchangeability, small size, low cost, long-term stability, and excellent sensitivity [8] makes the resistive sensors perfect candidates for humidity measurements.

However, the resistive humidity sensors’ performance, in terms of aspects such as sensitivity, response time, and recovery time, is strongly related to the sensing materials’ properties [9]. Therefore, a diversity of materials has been studied as the sensing layer within the design of humidity sensors: metal oxides semiconductors [10,11], polyelectrolytes [12,13], conducting polymers like poly(3,4-ethylenedioxythiophene–poly(styrene-sulfonate) [14] or polyaniline [15], and perovskites [16,17].

Carbon-based materials are intensively utilized as sensing layers in the manufacturing of humidity sensors. Among these materials, we can mention the following: carbon nanotubes and their nanocomposites [18,19], nanodiamonds and their nanocomposites [20,21], fullerenes and their derivatives [22,23], graphene [24,25], carbon nanofibers [26], carbon nanosheets and carbon nanohoneycombs [27], and oxidized carbon nano onions and their nanocomposites [28,29,30,31].

In the last years, two interesting nanocarbonic materials have received increasing attention regarding their utility as sensing layers in resistive humidity sensors: carbon nanohorns and graphene oxide.

Single-walled carbon nanohorns (SWCNHs), also known in the literature as nanocones, are carbon nanostructures with a particular morphology and were first reported by Iijima and co-workers in 1998 [32]. SWCNHs consist of sp^2^ hybridized carbon atoms that form a conical molecular architecture of 2–5 nm in diameter and 30–50 nm in length [33,34]. These structures have some outstanding characteristics: facile synthesis methods, availability of high-purity samples, high chemical and thermal stability, large specific surface area, good electrical conductivity, high porosity, and versatile covalent and noncovalent functionalization [35]. The SWCNHs have been widely explored for different applications such as the design and construction of fuel cells (suitable support materials for catalyst metal) [36], carrier material for drug delivery systems [37], the design and construction of solar thermal collectors [38], rechargeable batteries [39], gas storage media [40], and so forth. However, little information about the gas sensing properties of either pristine or functionalized SWCNHs or their nanocomposites is available. Sano et al. proposed a resistive sensor for ammonia and ozone detection at room temperature using SWCNHs as a sensing thin film. The authors demonstrated that the adsorption of O_3_ induced the decrease of the resistance carbonaceous sensing layer, whereas the adsorption of NH_3_ increased the resistance of the nanocarbonic thin film [41]. Suehiro et al. described the fabrication of a sensor for nitrogen dioxide, which uses SWCNH as a sensing layer. It was demonstrated that the SWCNHs aggregate behaved as a p-type semiconductor [42].

Different nanocomposites and nanohybrids based on oxidized carbon nanohorns (CNHox) were also used in the design and manufacturing of chemiresistive sensors for ethanol vapor detection [43,44].

Oxidized carbon nanohorns (CNHox), a hydrophilic type of functionalized carbon nanohorns, were recently introduced as a key sensing element in the design of resistive humidity sensors. The structure exhibited good RH sensitivity when varying RH from 0% up to 90%, either in humid nitrogen or in moist air [45,46].

CNHox’s nanocomposites with hydrophilic polymers, such as poly(ethylene glycol)-blockpoly(propylene glycol)-block-poly(ethylene glycol) (PEG-PPG-PEG) and polyvinylpyrrolidone (PVP), are also used as sensing layers for resistive monitoring of humidity. The resistive sensors based on these sensing films are characterized by a rapid response time, excellent stability, and good sensitivity, comparable to that of a commercially capacitive relative humidity sensor [47,48].

Nanohybrids such as CNHox-SnO_2_-PVP and CNHox-ZnO-PVP were used as sensing layers in resistive humidity sensor designs, using a metallic interdigitated transducer (IDT) structure deposited on Si/SiO_2_ structure. For both nanohybrid configurations investigated, the sensing layer’s conductivity decreases while the RH level increases. Two types of sensing mechanisms are identified and discussed [49,50].

Graphene oxide (GO), one of the most studied derivatives of graphene, has received increasing attention as a sensing layer for humidity detection [51,52,53,54]. Thanks to its oxygen functional groups (epoxy, phenolic, carbonylic, and carboxylic), GO is strongly hydrophilic and proton conductive. Moreover, GO has some outstanding advantages: low production cost, solubility in water and organic solvents, facile covalent and noncovalent functionalization, large-scale production, long-term stability, and easy processing. All these features qualify GO as an appropriate material for humidity monitoring [55].

This paper presents the RH sensing response of a resistive sensor employing a sensing layer based on a ternary nanocomposite comprising oxidized carbon nanohorns GO-CNHox-PVP at different w/w/w ratios. The experimental data were compared with the sensing data of the sensitive layers based on CNHox and PVP-CNHox binary mixture previously reported in the literature [45,47].

For the first time to our knowledge, the results demonstrate the relative humidity sensing capabilities of a GO-CNHox-hydrophilic polymer nanocomposite at different ratios when used in a resistive humidity sensor operating at a room temperature.

## 2. Materials and Methods

### 2.1. Materials

**Figure 1:**
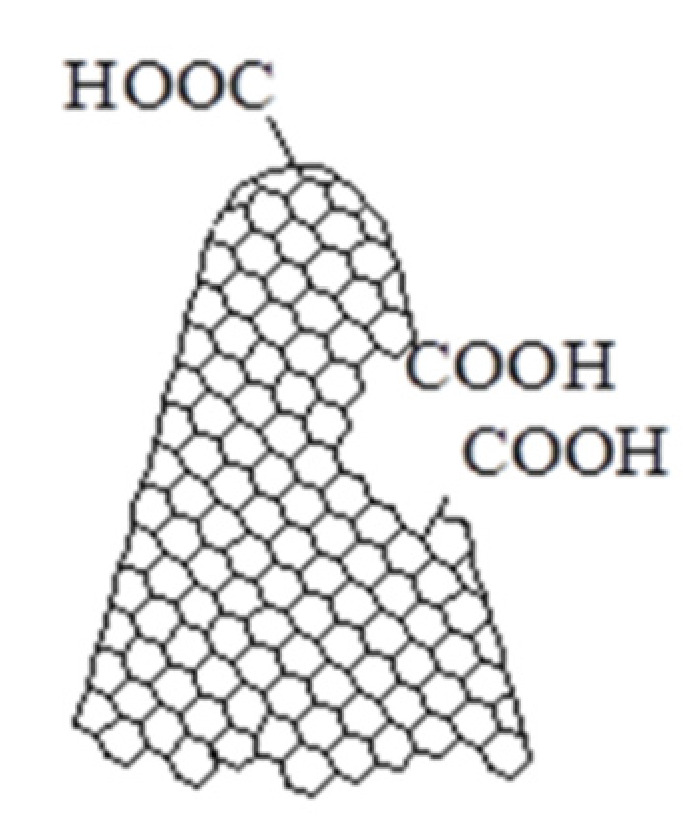
The structure of CNHox.

Powder of CNHox (with the structure depicted in Figure 1) was purchased from Sigma Aldrich (Redox Lab Supplies Com, Bucharest, Romania) and characterized by diameters between 2 nm and 5 nm (TEM), lengths between 40 nm and 50 nm, and specific surface area around 1300–1400 m^2^/g (BET). CNHox contains 10% graphite and has no metal contamination.

**Figure 2:**
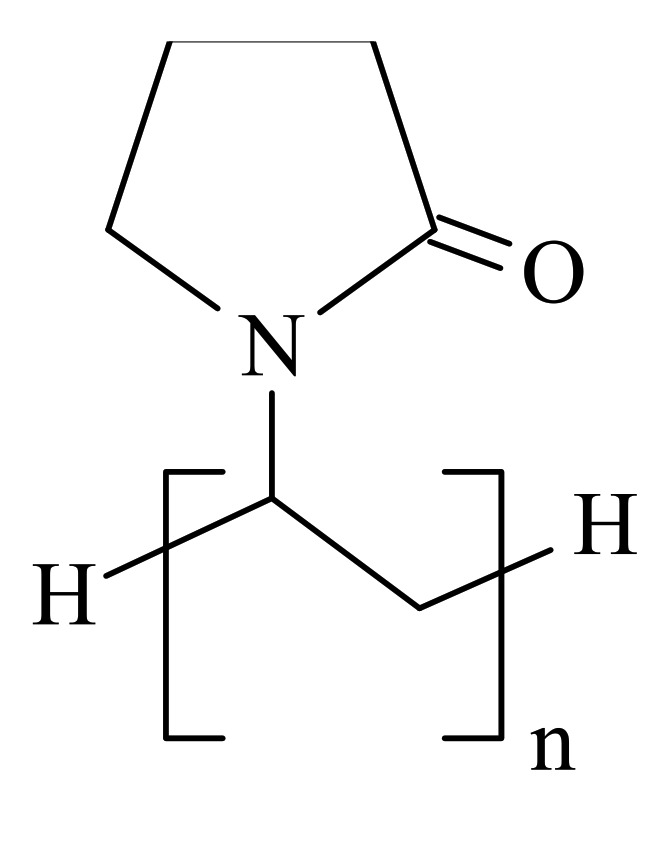
The structure of PVP.

**Figure 3:**
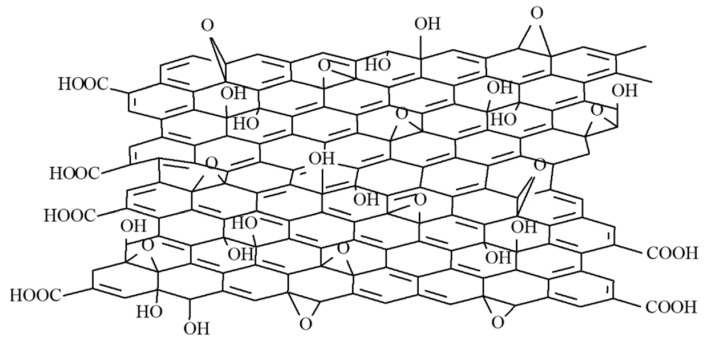
The structure of GO.

Polyvinylpyrrolidone (abbreviated as PVP, depicted in Figure 2, with an average mol wt 40,000), isopropanol (70% w/w in water), and graphene oxide (abbreviated as GO, depicted in Figure 3, 15–20 sheets, 4–10% edge-oxidized, 1 mg/mL dispersion in water) were also purchased from Sigma-Aldrich. No further purification of the agents was conducted before the experiments.

### 2.2. Methods

The surface topography of the sensing films based on the GO-CNHox-PVP ternary nanocomposite was investigated by scanning electron microscopy (SEM). For surface visualization, a field emission gun scanning electron microscope/FEG-SEM-Nova NanoSEM 630 (Thermo Scientific, Waltham, MA, USA.) (FEI), with superior low voltage resolution and high surface sensitivity imaging, was used.

The Raman spectra were collected at room temperature with a Witec Raman spectrometer (Alpha-SNOM 300 S, WiTec. GmbH, Ulm, Germany) using 532 nm as an excitation. The 532-nm diode-pumped solid-state laser has a maximum power 145 mW. The incident laser beam with a spot-size of about 1.0 µm was focused onto the sample with a 100× long-working distance microscope objective. The Raman spectra were recorded using a 20 s exposure time; the scattered light was collected by the same objective in back-scattering geometry with 600 grooves/mm grating. The calibration of the Raman systems was carried out using the 512 cm^−1^ Raman line of a silicon substrate which correspond to the longitudinal optical-transverse optical (LO-TO) phonon. The spectrometer scanning data collection and processing were carried out by a dedicated computer using WiTec Project Five software (WiTec Project Five 5.1, WiTec. GmbH, Ulm, Germany).

### 2.3. The Synthesis of the Ternary Nanocarbonic Materials-Based Nanocomposites Sensing Films

For the investigation of the relative humidity sensing capabilities of the nanocarbonic materials-based nanocomposites, the following chemical compositions of the sensing films were designed and tested: GO-CNHox-PVP (1/1/1), GO-CNHox-PVP (1/2/1), and GO-CNHox-PVP (1/3/1), all mass ratios (w/w/w).

We developed and followed a unique synthesis procedure for all three ternary compositions mentioned above. For example, the synthesis of the solid-state sensing films based on GO/CNHox/PVP = 1/1/1 as a mass ratio (w/w/w) is described below.

CNHox (0.1 g) was dispersed in 20 mL isopropanol and subjected to stirring in an ultrasonic bath for three hours at room temperature. PVP (0.1 g) was added to this dispersion, followed by stirring in the ultrasonic bath for two hours at room temperature. An amount of 0.1 g GO dispersion was added to the previous suspension, and continuous stirring was performed in the ultrasound bath for three hours at room temperature [56].

Finally, a two-step annealing process was performed as follows:

−  heating for 20 h at 80 ℃ under low pressure (2 mbar);
−  heating for 90 h at 110 ℃ under low pressure (2 mbar).

The sensing layer was obtained by depositing—using the drop-casting method—the dispersion over the IDT sensing structure, with the contact area being masked.

**Figure 4:**
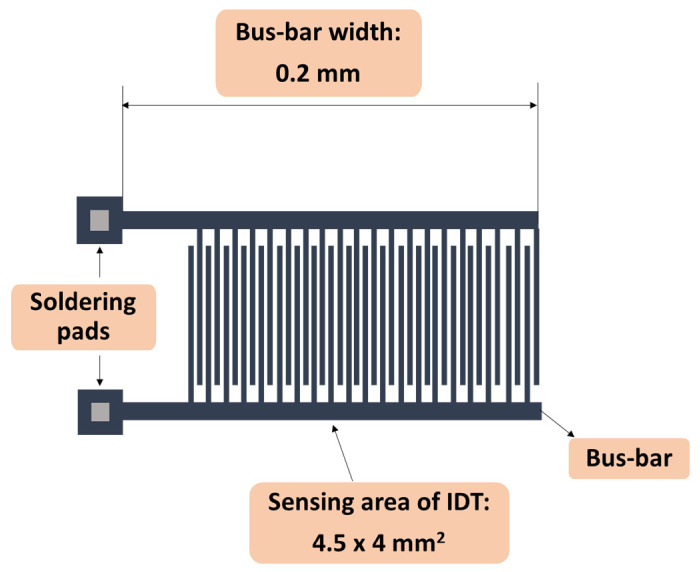
The layout of the (interdigitated) IDT sensing structure.

The sensing device consisted of a metallic interdigitated (IDT) dual-comb structure fabricated on a Si substrate (470 µm thickness), covered by a SiO_2_ layer (1 µm thickness) (Figure 4). The metal stripes of IDT were comprised of chromium (10 nm thickness) and gold (100 nm thickness). The digits’ width and spacing were equal to 10 microns, with a 0.6 mm separation between digits and the bus-bar.

The reference humidity sensor used during the experiments was an industrial grade capacitive sensor, with a typical accuracy of ± 2% in the humidity range 0–100% RH and with a working temperature domain of 0–900 ℃ (for temperature).

**Figure 5:**
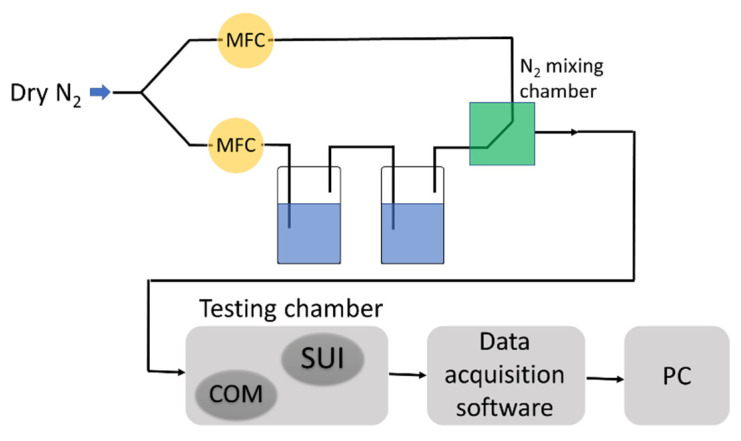
Experimental setup employed for RH measurements (MFC—mass flow controller, SUI –sensor under investigation, COM—commercial sensor).

We performed the relative humidity sensing measurements in a special testing bench (Figure 5), where for varying the relative humidity in the testing chamber from 0% to 100% RH, the dry nitrogen was purged through two recipients in series containing deionized water. The humidity in the testing chamber (the size of the chamber was 10 × 8 × 4 cm^3^) was changed by mixing dry nitrogen passing through the containers with deionized water in different ratios. In the mixing chamber (the green square in Figure 5), the gases flowing on both paths formed a homogenous mixture that passed to the testing chamber. The chamber accommodated a tandem of sensors: our resistive sensing structure (depicted as SUI—sensor under investigation), which uses GO-CNHox-PVP as a sensing layer, and a capacitive relative humidity sensor, commercially available (COM—commercial sensor). The commercial sensor used in the experiment was a Sensirion Digital Humidity Sensor SHT4x sensor. The capacitive sensor was used to double-check the relative humidity level indicated by the mass flow controller (MFC) system. Mass flow controllers 4850 from Brooks Instruments with flow range 50 sccm–40 slpm, accuracy 3.0% FS, response time 300 ms were used. By positioning the two devices close to each other and to the gas inlet, both were exposed to the identical gas flow (dry nitrogen) to provide quasi-similar experimental conditions and thus lead to reliable conclusions. 

The relative humidity levels in the mixing chamber were obtained by mixing dry nitrogen with specific gas wet volumes, supplied by the bubblers. The total mixed gas flow in the testing chamber was always kept constant, i.e., one liter per minute. Each humidity jump took about 300 s and sensors’ responses were measured continuously, while the gas flow was adjusted periodically so that the humidity increased and the flow remained constant. Once the required RH level was achieved, which was checked by the mass flow controller, the gas flow was maintained constant up to 300 s. This procedure was applied for all the recorded points. The gas flow rates were established in a previous experiment with the same experimental setup, but with the capacitive sensor (commercial sensor) only. Thus, experimentally, an answer for the relative humidity according to the Equation (1) was achieved.

(1) $$\frac{V_{humid\;nitrogen}}{V_{dry\;nitrogen\;}+V_{humid\;nitrogen}}\cdot 100≅RH\%$$

A Keithley 6620 current source (Keithley Instruments GmbH, Germering, Germany), ensuring a current variation between 0.01–0.1 A was employed; the data were collected and analyzed with a PicoLog data logger (PICO Technology, Neots, Cambridgeshire, UK). All of the measurements were recorded at constant room temperature.

Test conditions for the proposed experimental setup were not always identical. We tried to make identical jumps of humidity in the testing room, but there were small differences as shown by the commercial sensor (reference sensor). This was the reason for using both sensors in the testing room, the commercial one, whose answer was accepted as a reference answer and the investigated sensor. The idea of the experimental setup was to be able to track the response of the investigated sensor with the reference sensor in order to find out if the sensor is suitable as a resistive humidity sensor.

## 3. Results and Discussion

**Figure 6:**
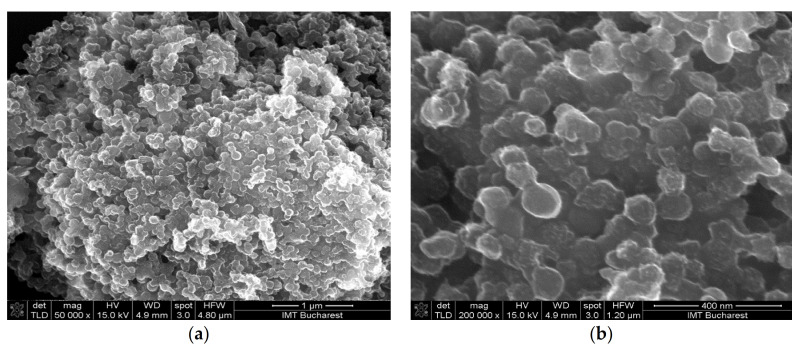
Scanning electron micrographs of the GO/CNHox/PVP at 1:1:1 w/w/w ratio: (**a**) ×50,000 magnification; (**b**) ×200,000 magnification.

**Figure 7:**
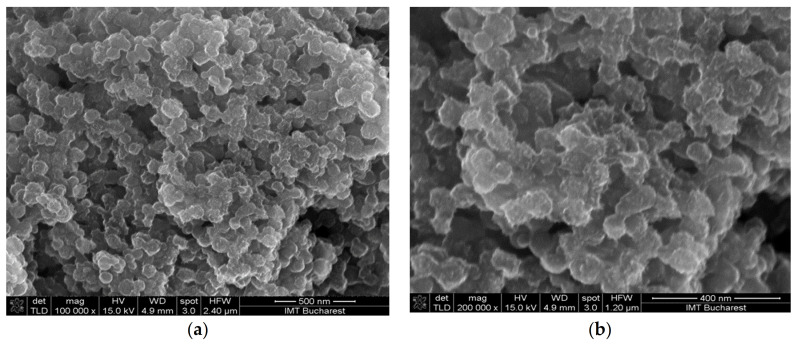
Scanning electron micrographs of the GO/CNHox/PVP at 1:2:1 w/w/w ratio: (**a**) ×100,000 magnification; (**b**) ×200,000 magnification.

**Figure 8:**
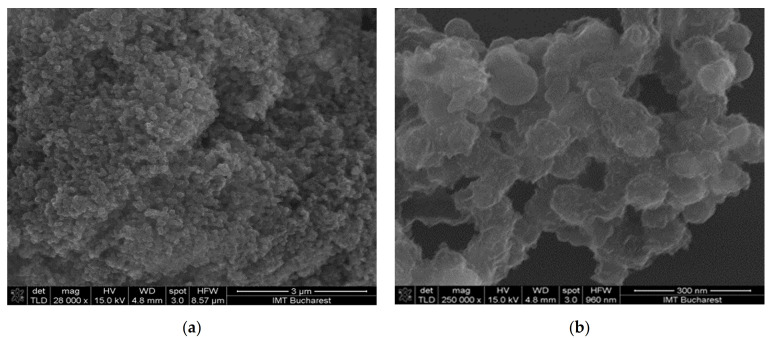
Scanning electron micrographs of the GO/CNHox/PVP at 1:3:1 w/w/w ratio: (**a**) ×28,000 magnification; (**b**) ×250,000 magnification.

The surface morphology of the deposited films was relatively homogenous in all cases (Figure 6, Figure 7 and Figure 8). The hydrogen bond, π-π stacking interactions between graphene oxide and CNHox, and film-forming properties of PVP were, most probably, responsible for these results [57,58].

At the same time, the interaction between the two nanocarbon materials with each other and with PVP was proven using Raman spectroscopy. 

**Figure 9:**
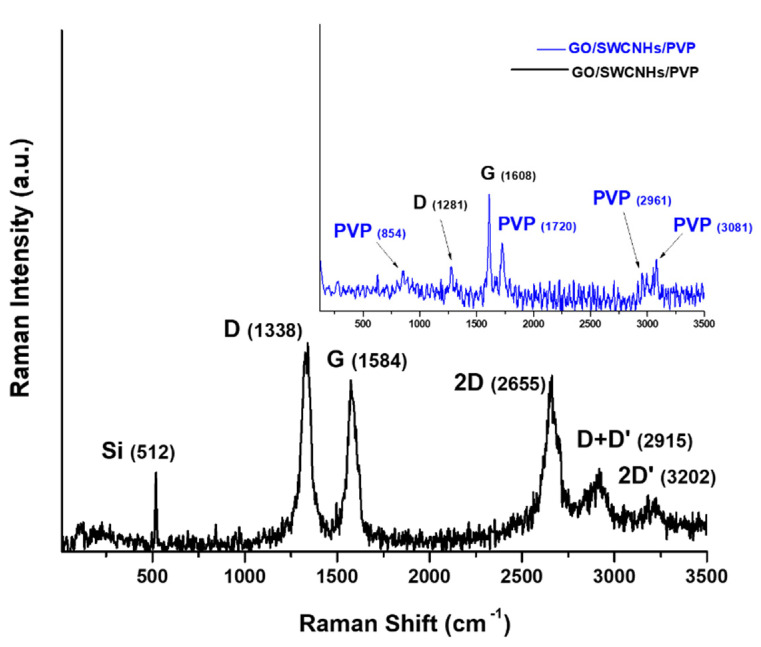
Raman spectra of solid-state films of GO/CNHox/PVP 1/1/1 (mass ratio) deposited on silicon substrate (top inset—the recorded Raman spectra from which the silicon response was subtracted).

Figure 9 presents the recorded Raman spectra for the GO/CNHox/PVP 1/1/1 nanocomposite on silicon (black color), while the top inset represents the recorded Raman spectra from which the silicon response was subtracted.

The recorded spectrum exhibits the typical D (~1338.8 cm^−1^), G (~1584.1 cm^−1^), D’ (1601 cm^−1^), and second-order bands of 2D and D + D’ band localized at 2655.5 and 2915.9 cm^−1^ which are characteristic for carbon nanomaterials. The Raman band from about 3202.7 cm^−1^ may be associated with the so-called 2D’ band and this may come from the vibration spectrum of graphite, the most important impurity from oxidized carbon nanohorns [43].

The very high intensity of the peak located at ≅ 520 cm^−1^ is associated with the silicon substrate [59]. Moreover, the well-known peaks belonging to PVP (854, 1429, 1665, 2925, and 2997 cm^−1^) [60] overlapped with the bands of nanocarbonic components. The Raman band from about 920 cm^−1^, which is not assigned in the spectrum of the carbonaceous nanocomposite, may come from the overlapping of the Raman bands of the silicon substrate and PVP [43]. Some of the small Raman shifts of the peaks associated to nanocarbonic materials [45] are a consequence, most probably, of the multiple chemical interactions such as stacking interactions, hydrogen bond between oxidized carbon nanohorns, graphene oxide, and polyvinylpyrrolidone.

The relative humidity monitoring capability of each carbonaceous nanocomposite-based thin film was explored by applying a current between the two electrodes and measuring the voltage difference when varying the RH from 0% to 100%. For the simplicity of our description, we use the folowing abbreviations:

Sensor 111—resisitive sensor which employed sensing layer based on GO/CNHox/PVP at 1/1/1 ratio (w/w/w);
Sensor 121—resisitive sensor which employed sensing layer based on GO/CNHox/PVP at 1/2/1 ratio (w/w/w);
Sensor 131—resisitive sensor which employed sensing layer based on GO/CNHox/PVP at 1/3/1 ratio (w/w/w);

An important characteristic of these devices is low power consumption, below 2 mW. The commercial sensor used in our experiments had a power consumption of up to 5 mW.

The behavior of all three manufactured sensors is presented below.

The resistance of the ternary nanocarbonic materials-based sensing layer increased when RH increased in the range from 0% to 100% RH and was measured with 10% RH steps.

**Figure 10:**
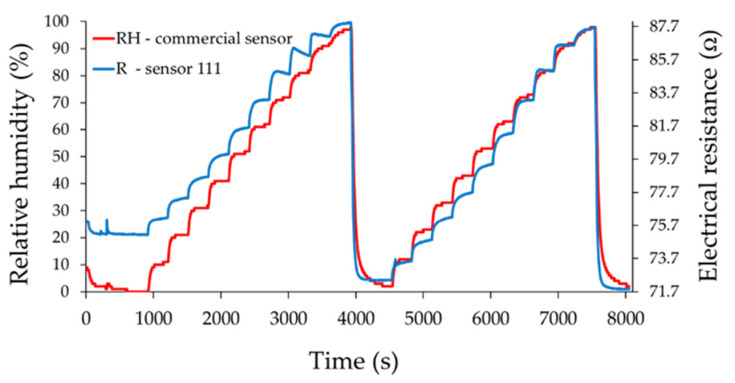
The response of sensor 111 (“R curve”—blue) as a function of time for two measurement cycles, when relative humidity was increased in ten steps from 0% RH to 100% RH; “RH curve—red” shows the similar characteristic measured for a commercial, industrial sensor.

**Figure 11:**
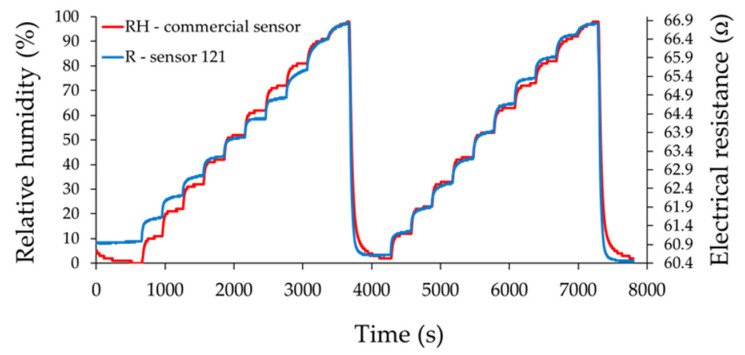
The response of the sensor 121 (“R curve”—blue) as a function of time for two measurement cycles, when relative humidity was increased in ten steps from 0% RH to 100% RH; “RH curve—red” shows the similar characteristic measured for a commercial, industrial sensor.

**Figure 12:**
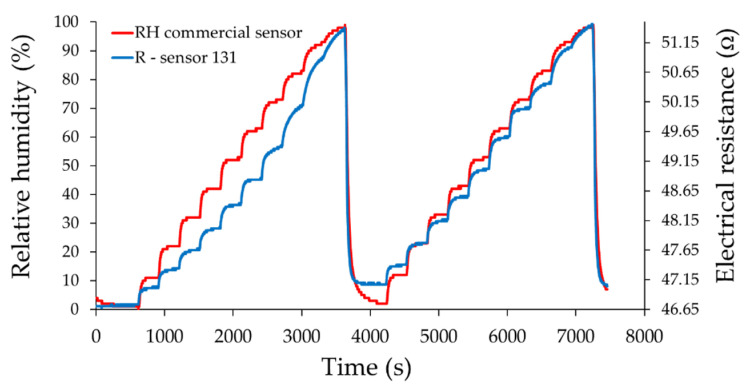
The response of the sensor 131 (“R curve”—blue) as a function of time for two measurement cycles, when relative humidity was increased in ten steps from 0% RH to 100% RH; “RH curve—red” shows the similar characteristic measured for a commercial, industrial sensor.

From Figure 10, Figure 11 and Figure 12, the drift of the sensor when returning to the “0” point of relative humidity is noticeable. The measured resistance of the sensing layer at 0% RH, between the measurement cycles, had approximately the same value when sensor 121 was used. 

The most plausible explanation is related to the presence of mass ratio graphene oxide-CNHox-PVP in the ternary nanocomposite. Due to a strong hydrophilic character, an increased content of GO in the composition of the ternary nanocomposite could yield to the generation of water clusters, with detrimental effects from the perspective of drift. An increased content of CNHox (more hydrophobic than GO and PVP) could avoid this drawback. On the other hand, decreasing the content of GO in the ternary nanocomposite, the bundles of oxidized carbon nanohorns cannot be dispersed in some extent to avoid capillary condensation of water.

**Figure 13:**
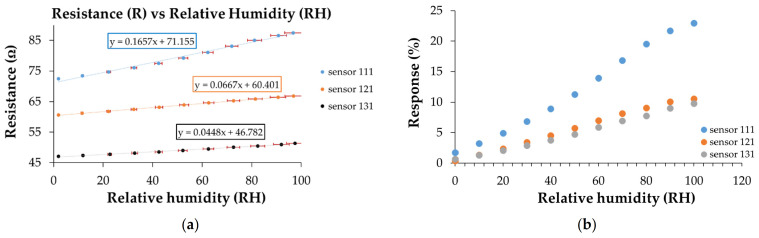
(**a**) The transfer function, in the second run, of the sensors in humid nitrogen (RH = 0–100%); (**b**) the humidity sensing response (%) varies with the amount of deposited CNHox.

Moreover, it was observed that the resistance of the sensitive layer for the RH value “0” decreased with the increase of the CNHox content in the nanocomposite. This is a natural behavior because CNHox is a nanocarbonic material with high conductivity, and increasing its concentration leads to a decrease of the electrical resistance of the nanocomposite. Figure 13a,b presents resistance and % response vs. RH values recorded by the commercial sensor after the recovery of the sensing layer in the second run from 0% to 100% RH. 

Figure 13a presents the transfer function between the sensing structures’ electrical resistance (measurable output) and the % RH measured in the testing chamber (the measurable input). The overall linearity of the ternary nanocarbonic materials-based resistive sensors in humid nitrogen when varying RH from 0% to 100% (measured with humidity accuracy of ± 3.5%) fits well with the experimental data, as shown in Figure 13a.

The sensor response is defined as:

(2) $$Response\left(\%\right)=\frac{\Delta R}{R_{dry\;nitrogen}}\times 100\%=\frac{R_{humid}-R_{dry\;nitrogen}}{R_{dry\;nitrogen}}\times 100\%$$

where *R_humid_* and *R_dry nitrogen_* are the measured resistances exposed to humid conditions and dry nitrogen, respectively. The response of the sensors with the composite sensing layer when exposed to different RH levels at room temperature is plotted in Figure 13b. From this graphical representation, we can see that the calculated % response increased for all the sensing layers with RH, and that sensor 111 had the best % response when compared with the other two sensors, sensor 121 and sensor 131.

For the comparison of the sensing performances of the RH resistive sensors, with different initial resistance values, a relative sensitivity (S_r_) to relative humidity variations (RH) was defined. 

The overall increasing resistance of the ternary nanocomposite-based sensing layer was in line with previous results regarding the RH resistive sensing capabilities of CNHox and its nanocomposites.

The sensitivity of the sensor was defined as follows:

(3) $$S=\frac{\Delta R_{x}}{\Delta RH_{x}}=\frac{R_{x}-R_{0}}{RH_{x}}$$

where *R_x_* is the resistance of the sensitive layer measured in the test chamber for the *RH_x_* value indicated by the commercial sensor (measured with ± 2% accuracy as the producer indicates it). *R*_0_ is the estimated resistance from the graph calibration line resistance = f (relative humidity) by extrapolation for the value at RH 0%. The comparison is summarized in Table 1.

CNHox and CNHox-PVP-based sensing layers were proven suitable for RH resistive monitoring, but the latter proved to have a superior performance. Thus, the beneficial effect of the PVP addition in terms of increasing the sensitivity of the sensor for almost the entire relative humidity range was demonstrated [45,47]. However, the synthesized ternary nanocomposites GO-CNHox-PVP-based sensing layer exhibited a better sensitivity and linearity in comparison with the binary nanocomposite CNHox-PVP [47]. The p-type semiconducting properties of GO and ability to disperse bundles of oxidized carbon nanohorns were the main responsible factors leading to increasing sensor performance (present work).

**Figure 14:**
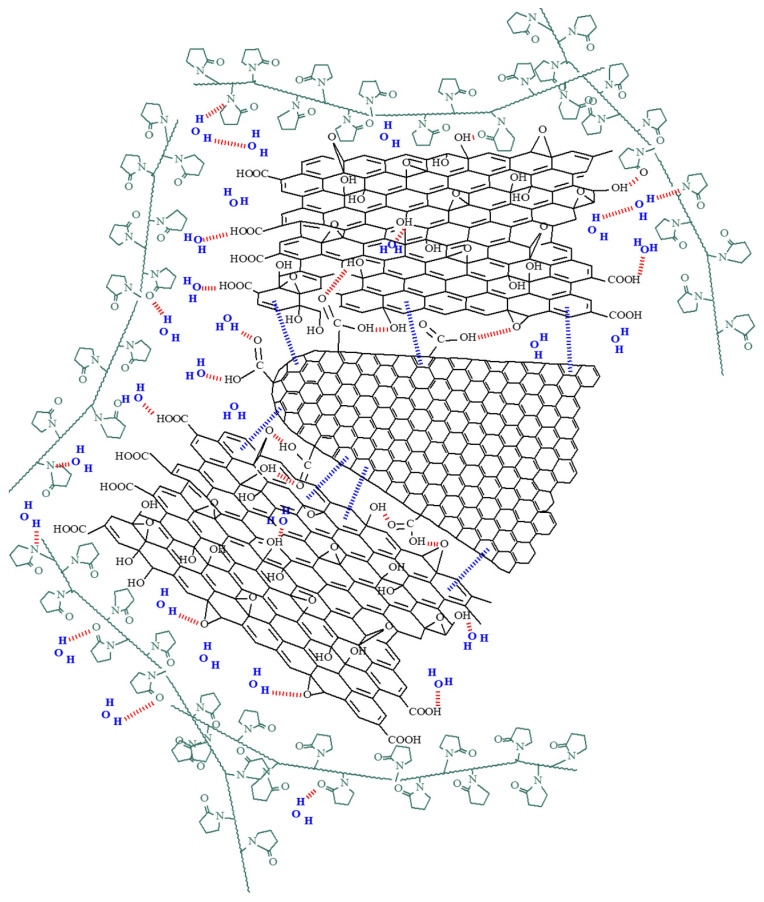
Possible structure for supramolecular aggregate generated by CNHox and GO through hydrogen bonds and pi-pi stacking interaction. Interaction of water molecules with these supermolecules is the core of the humidity sensing.

The results presented in Table 1 may seem rather unexpected and curious at first glance. Given the principle of sensing and the fact that oxidized carbon nanohorns are the major contributor to the sensing layer conductivity, an increase in sensitivity would be expected with increasing CNHox concentrations. On the contrary, increasing the concentration of carbon nanohorns leads to a decrease of S_r_. The most plausible explanation is related to the presence of graphene oxide. Carbon nanohorns and their functional derivatives generate large aggregates (a possible structure is presented in Figure 14). The higher the concentration of CNHox, the more compact and the lower the specific surface area of the aggregates. Through intermolecular hydrogen bonds and π–π stacking interaction, GO can act as suitable dispersants for CNHox and is able to redisperse the bundles of oxidized carbon nanohorns. Thus, graphene oxide enhances surface area and improves humidity sensing. This comparative analysis suggests that there is an optimum ratio of the CNHox/GO, that should be used for maximizing the sensitivity of ternary nanocomposites toward moisture. This is a good starting point for further CNox/GO ratio optimization.

For a sensor operation with the sensing film kept at room temperature (RT), it is important to evaluate the response and recovery time. Both adsorption and desorption of water molecules to and from the polymer matrix may be very slow under such RT circumstances. 

**Figure 15:**
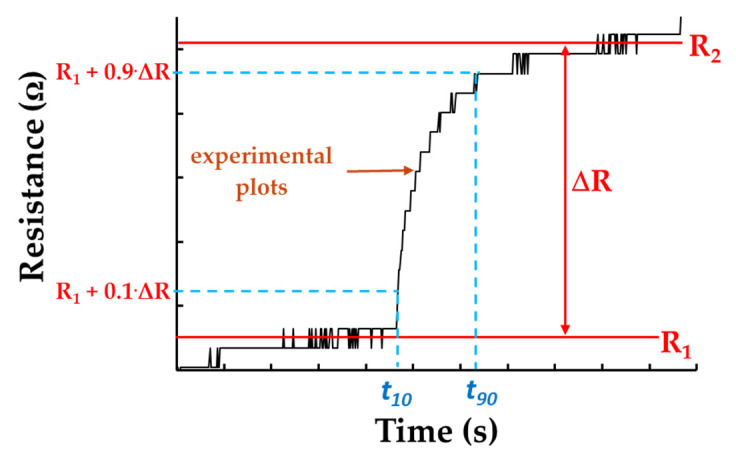
Example for calculation of response time.

The response time ($t_{r}$) of the humidity sensor represents a fundamental parameter in order to evaluate the overall performance of the sensor; if *R(t)* is the response of the device in time, the $t_{r}$ can be evaluated as:

(4) $$t_{r}=t_{90}-t_{10},$$

where $t_{90}$ and $t_{10}$ represent the moments in time where the response *R(t*) reaches 90% and 10%, respectively, from the total variation of the sensor’s resistance as a result of a change in the value of the RH [45]. Similarly, the recovery times were calculated (see the example in Figure 15).

**Figure 16:**
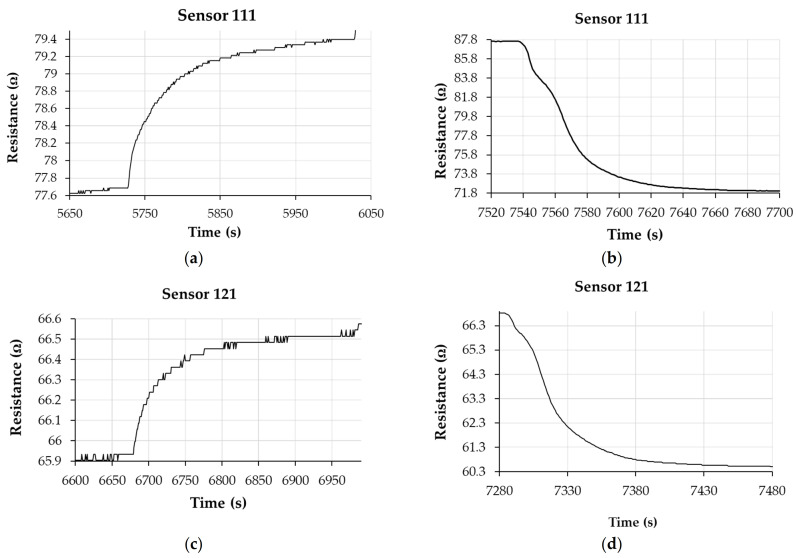

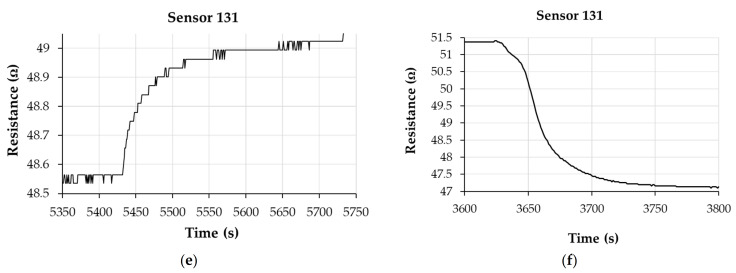
The response and recovery times of the chemiresistive 111, 121, and 131 humidity sensors with sensing films kept at room temperature where (**a**) is the response time and (**b**) is the recovery time for sensor 111; (**c**) is the response time and (**d**) is the recovery time for sensors 121; (**e**) is the response time and (**f**) is the recovery time for sensor 131. Response time was measured for the case when relative humidity was increased from 40% to 50%, while the recovery time was measured from 100% to 0% RH (clean dry nitrogen).

In Figure 16, the response and recovery times of the chemiresistive test structures containing sensing films based on sensors 111, 121, and 131 are shown for the case when relative humidity was increased from 40% to 50%, for response time, and from 100% to 0% RH (clean dry nitrogen) for the recovery time.

**Figure 17:**
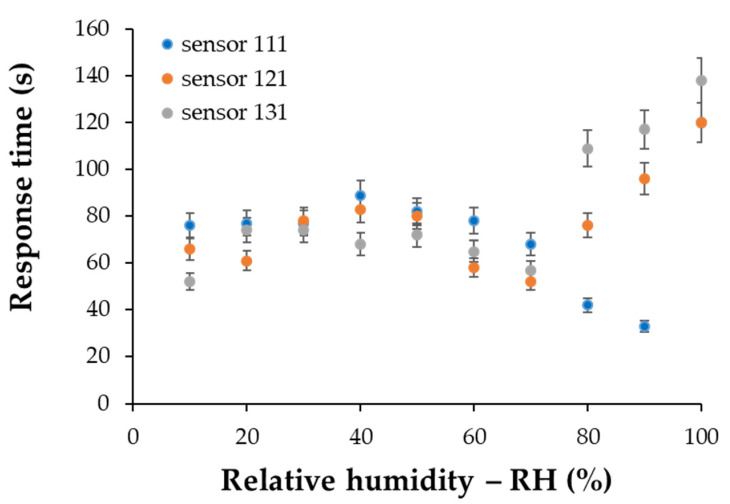
Response time vs. relative humidity (RH %) for sensor 111, sensor 121, and sensor 131.

The values of response times for the commercial sensor were in the range of 40–90 s, the higher values being recorded for RH values of over 50%. Comparing the response of the investigated sensors, we observed that sensor 111 showed values of response times in the range 40–90 s, while sensors 121 and 131 had response times between 50–100 s and 50–110 s, respectively (Figure 17).

As an example, for the RH variation from 40% to 50%, the response times of sensors 111, 121, and 131 from Figure 10, Figure 11 and Figure 12 decreased from 82 to 80 s and 72 s, respectively. However, it was observed that for lower values of humidity (up to 50% RH), the response time was longer for almost all sensors compared to the commercial sensor, and this result should be further understood. On the other hand, for sensor 121 and sensor 131, longer response times were obtained at RH levels higher than 50% compared with values for response times when RH values were below 50%, and this result can be explained by the decreasing number of actives sites at high relative humidity, as follows. Water molecules penetrate the hydrophilic sensing layer based on GO/CNHox/PVP. Due to the presence of hydrophilic polymer (PVP) and oxygen functional groups (carboxylic, epoxy, phenolic, carbonylic) in CNHox and GO, the nanocomposite film adsorbs a substantial amount of water. Finally, some water molecules can condensate in the vicinity of hydrophilic groups and block the active sites. On the other hand, our sensors had a shorter recovery time with respect to the commercial sensor when the RH values were decreased from 100% RH to 0% RH, as shown in Table 2. 

### Analysis of Sensing Mechanism

Each component of the ternary nanocomposites used for the resistive monitoring of RH had a well-defined role. CNHox exhibited outstanding properties such as increased conductivity (p-type semiconductor), high uniformity, hydrophilicity high purity (no metallic compound), and a large surface area (1300 to 1400 m^2^/g). All these features make CNHox a promising candidate for monitoring the RH at room temperature.

GO shows a lot of advantages such as hydrophilic properties, good charge carrier, low-cost fabrication, long-term stability, and excellent dispersant for carbon nanohorns. Moreover, GO is a p-type material and exhibits a decrease in electrical conduction when exposed to moisture [61,62].

Polyvinylpyrrolidone (PVP) is a hydrophilic polymer with excellent binder properties. It is important to mention that PVP is a hygroscopic, electrically insulating polymer, absorbing up to 25% moisture at 75%. Therefore, water is adsorbed into the bulk of the sensing layer, not only at the surface of the film.

Given the features of the above-mentioned materials, three distinct sensing mechanisms can be discussed.

The first approach takes into account the dissociation of water. The adsorbed water molecules on GO and CNHox hydrophilic surface may dissociate to H^+^ and OH^−^ ions. The protons generated by the water dissociation process may tunnel from one water molecule to another through hydrogen bonding, increasing the overall electrical conductivity of the sensitive film [52].

The second mechanism considers the fact that GO and CNHox are p-type semiconducting materials. When interacting, the water molecules donate their electron pairs, thus decreasing the number of holes in both nanocarbonic materials. Therefore, the humidity sensing layer based on the ternary nanocomposite becomes more resistive. Several results reported in the literature are in agreement with this statement [63].

Last but not least, PVP is a dielectric polymer with hydrophilic properties, swells due to interaction with water molecules, and has a negligible change in resistance in response to variation in RH as shown for the presented composites [64].

As a consequence, the distance between GO and CNHox moieties increases, and electrically percolating pathways are reduced. Again, the sensing layer becomes more resistive [62].

As we can see, the interaction of the sensing layer with moisture has two opposing effects on resistance. We presume that the effect of the p-type semiconductor behavior of CNHox and GO correlated with the swelling of PVP dominates and leads to the overall increasing resistance of the sensing layer.

In fact, the overall increasing resistance of the ternary nanocomposite-based sensing layer are in line with the previous results regarding the RH resistive sensing capabilities of CNHox alone and CNHox-PVP nanocomposite. Both sensing layers are suitable for RH resistive monitoring, but the latter proved to have a superior performance. Thus, the beneficial effect of the PVP addition in terms of increasing the sensitivity of the sensor for almost the entire relative humidity range was demonstrated [45,47]. All obtained ternary nanocomposites-based sensing layers exhibited a better sensitivity and linearity in comparison with binary nanocomposite CNHox-PVP [47]. The p-type semiconducting properties of GO and its ability to disperse bundles of oxidized carbon nanohorns are the main responsible factors that lead to increasing sensor performance.

## 4. Conclusions

This paper reports the RH sensing response of a resistive sensor employing a sensing layer based on a ternary nanocomposite comprising graphene oxide-oxidized carbon nanohorns-polyvinylpirrolidone (GO-CNHox-PVP), at 1/1/1, 1/2/1, and 1/3/1 w/w/w ratio. The sensors have a room temperature response comparable to that of a commercially humidity sensor, characterized by a rapid response, excellent linearity, good sensitivity, and a good recovery time. A detailed analysis in order to obtain the transfer functions, response, and recovery times of all manufactured sensing devices was performed. While the structures with GO/CNHox/PVP at 1/1/1 ratio (w/w/w) had the best performance in terms of relative sensibility, response time, and recovery time, structures with GO/CNHox/PVP at 1/2/1 ratio (w/w/w) had the best linearity. The key role of each component of the ternary nanocomposites was explained based on their chemical structure and their physical and chemical properties. Special attention was devoted to the complex interaction (π-π stacking and hydrogen bonding) between GO and CNHox which seems to be responsible for the better performance of the sensor. Three sensing mechanisms were considered and analyzed. The effect of the p-type semiconductor behavior of CNHox and GO correlated with swelling of PVP dominated and led to the overall increasing resistance of the sensing layer.

The low power consumption of these devices, below 2 mW, and the sensing performances at room temperature, as well as the simplicity of the sensor manufacturing are the most important advantages of presented devices.

## 5. Patents

*Ternary nanocomposite for the relative humidity resistive sensor and method for its manufacture*, Patent Application No. A/00585, 23.09.2019, OSIM, Romania, Bogdan-Catalin Serban, Octavian Buiu, Viorel Avramescu, Cornel Cobianu, Roxana Marinescu.

## Figures and Tables

**Table 1:** Sensitivity of GO-CNHox–PVP compared with previously tested sensing layers [45,47].

Sensing Layer	Sensitivity
CNHox [45]	0.013–0.021
PVP + CNHox 1/1 [47]	0.020–0.058
PVP + CNHox 2/1 [47]	0.017–0.025
GO-CNHox–PVP 1/1/1	0.150–0.200
GO-CNHox–PVP 1/2/1	0.063–0.070
GO-CNHox–PVP 1/3/1	0.043–0.051

**Table 2:** Comparison between recovery time for the developed sensors and the commercial sensor.

Recovery Time (s)						
	Commercial Sensor	Sensor 111	Commercial Sensor	Sensor 121	Commercial Sensor	Sensor 131
Recovery time	121	62	111	73	114	73

## Data Availability

The data presented in this study are available within the present article.
